# A randomized controlled trial on the effects of blue-blocking glasses compared to partial blue-blockers on sleep outcomes in the third trimester of pregnancy

**DOI:** 10.1371/journal.pone.0262799

**Published:** 2022-01-28

**Authors:** Randi Liset, Janne Grønli, Roger E. Henriksen, Tone E. G. Henriksen, Roy M. Nilsen, Ståle Pallesen

**Affiliations:** 1 Department of Psychosocial Science, Faculty of Psychology, University of Bergen, Bergen, Norway; 2 Department of Biological and Medical Psychology, Faculty of Psychology, University of Bergen, Bergen, Norway; 3 Faculty of Health and Social Sciences, Western Norway University of Applied Sciences, Bergen, Norway; 4 Division of Mental Health Care, Fonna Local Health Authority, Valen Hospital, Valen, Norway; 5 Norwegian Competence Center for Sleep Disorders, Haukeland University Hospital, Bergen, Norway; 6 Optentia, The Vaal Triangle Campus of The North-West University, Vanderbijlpark, South-Africa; University of Pittsburgh Graduate School of Public Health, UNITED STATES

## Abstract

**Objective:**

Sleep disturbances are common in pregnancy. Blocking blue light has been shown to improve sleep and may be a suitable intervention for sleep problems during pregnancy. The present study investigated the effects of blue light blocking in the evening and during nocturnal awakenings among pregnant women on primary sleep outcomes in terms of total sleep time, sleep efficiency and mid-point of sleep.

**Methods:**

In a double-blind randomized controlled trial, 60 healthy nulliparous pregnant women in the beginning of the third trimester were included. They were randomized, using a random number generator, either to a blue-blocking glass intervention (n = 30) or to a control glass condition constituting partial blue-blocking effect (n = 30). Baseline data were recorded for one week and outcomes were recorded in the last of two intervention/control weeks. Sleep was measured by actigraphy, sleep diaries, the Bergen Insomnia Scale, the Karolinska Sleepiness Scale and the Pre-Sleep Arousal Scale.

**Results:**

The results on the primary outcomes showed no significant mean difference between the groups at posttreatment, neither when assessed with sleep diary; total sleep time (difference = .78[min], 95%CI = -19.7, 21.3), midpoint of sleep (difference = -8.9[min], 95%CI = -23.7, 5.9), sleep efficiency (difference = -.06[%], 95%CI = -1.9, 1.8) and daytime functioning (difference = -.05[score points], 95%CI = -.33, .22), nor by actigraphy; total sleep time (difference = 13.0[min], 95%CI = -9.5, 35.5), midpoint of sleep (difference = 2.1[min], 95%CI = -11.6, 15.8) and sleep efficiency (difference = 1.7[%], 95%CI = -.4, 3.7). On the secondary outcomes, the Bergen Insomnia Scale, the Karolinska Sleepiness Scale and the Pre-Sleep Arousal Scale the blue-blocking glasses no statistically significant difference between the groups were found. Transient side-effects were reported in both groups (n = 3).

**Conclusions:**

The use of blue-blocking glasses compared to partially blue-blocking glasses in a group of healthy pregnant participants did not show statistically significant effects on sleep outcomes. Research on the effects of blue-blocking glasses for pregnant women with sleep-problems or circadian disturbances is warranted.

**Trial registration:**

The trial is registered at ClinicalTrials.gov (NCT03114072).

## Introduction

Sleep changes occur throughout pregnancy. Pregnant women typically experience increased need for sleep in the first trimester and increased sleep disturbances during the third trimester [1]. By gestational week 40, as many as 75–98% report multiple nocturnal awakenings [2–5].

Insomnia is the most frequent sleep disorder in pregnancy reported by 62% of pregnant women, which is significantly higher than found in the general population (10–15%) [1, 6, 7]. Insomnia manifests as difficulties falling asleep, maintaining sleep, or early morning awakenings, occurring for at least three nights per week [8]. Several hormonal and mechanical influences can cause insomnia during pregnancy, including nocturia (a frequent need to rise and urinate at night), dyspnea (shortness of breath), nasal congestion, muscular aches and pelvic pains, fetal activity, leg cramps as well as reflux [1].

Sleep disturbances during pregnancy are associated with adverse pregnancy outcomes including preeclampsia, elevated serum glucose, depression, prolonged labor, cesarean birth, intrauterine growth restriction and preterm birth [1, 9, 10]. Insomnia is also a significant risk factor for development of depression during the prenatal and postpartum period, in particular if it debuts in the third trimester of pregnancy [6].

Effective treatments for sleep disturbances, which are documented as safe for use during pregnancy are currently lacking [11]. Hypnotic medications such as benzodiazepines, some antidepressants, melatonin and antihistamines are available as for the general population. However, there is a dearth of research on the effects and potential side-effects of such medications, especially for the fetus, when used during pregnancy [12]. Still, some evidence suggest that such drugs are linked with adverse neonatal outcomes [13]. Hence, medication is not a recommended first-line treatment for sleep problems during pregnancy [1, 11]. Non-pharmaceutical treatments such as sleep hygiene counselling or cognitive behavior therapy for insomnia [14] may have a treatment potential, but the evidence for a clinically significant effect on the pregnant population is scarce [1, 11, 12]. In addition, such treatments are relatively time-consuming, costly, and often not readily available. Hence, effective treatment options should be explored.

One assumingly harmless intervention would be to reduce evening and night light exposure, promoting natural mechanisms for sleep initiation and maintenance. Even though studies on acute alerting effects of artificial light exposure in the evening and night have used small sample sizes which make it difficult to draw definitive conclusions [15], light has been shown to reduce evening and night sleepiness [15–18]. Moreover, a meta-analysis shows that evening light is associated with later bedtime and shorter sleep time [19]. Light at night can reduce the quality of sleep in terms of repeated awakenings [20], interrupting sleep [21], and reduce quality of the deep, restorative sleep [16]. Conversely, behavioural interventions for sleep problems in terms of evening light avoidance seem to have highest effectiveness [21].

Light is known to be the principal environmental factor (zeitgeber) regulating circadian rhythms [22]. Darkness allows production of melatonin, a hormone which regulates the circadian rhythm, and facilitate sleep. Non-visual effects of light are conveyed by sensitive special photoreceptors of the retina, intrinsically photoresponsive retinal ganglion cells (ipRGCs) which project to the suprachiasmatic nucleus (SCN) of the hypothalamus [22]. IpRGCs are most sensitive to the frequencies between 446 and 484 nm [23, 24]. Evening and nighttime exposure to wavelength shorter than 530 nm (blue light), suppresses the melatonin production, delay circadian rhythms and inhibit sleep [25–27].

Studies investigating the relationship between light exposure and sleep in pregnant women are limited. One study found that light exposure at night was associated with reduced sleep duration in the first and third trimester [28]. A more recent study showed that evening light exposure in pregnant women was related to shorter total sleep time and earlier midpoint of sleep as measured by actigraphy [29]. There is a dearth of knowledge regarding the burden light exposure might have on pregnant women and if blocking such light improves sleep in this population.

Artificial light-sources such as smart-phones, tablet, computers and TV often have illumination with a relative high amount of blue light and some studies have shown that such illumination increases alertness, delay onset of the deep restorative sleep stage and suppresses the melatonin production [16, 30]. Glasses that block blue light (BB-glasses) have accordingly shown to prevent alertness caused by blue-light emitting screens [31, 32].

Studies have further shown that use of BB-glasses are able to relieve sleep disturbances [33], particular with individuals with insomnia [32, 34, 35], bipolar disorder [36] and attention-deficit hyperactive disorder (ADHD) [37]. These treatment effects have further been attested to by a recent review [38].

In terms of fertile women BB-glasses may speed recovery for postpartum depression sufferers [31]. To our knowledge, no previous studies have investigated the effect of BB-glasses on sleep outcomes during pregnancy.

Blue blocking glasses have previously been shown to produce rapid effects on sleep and activation [31, 35–37], and exploring nonpharmacologic treatment of insomnia in pregnancy is warranted [11, 13].

The present study initiates new research by investigating the effects of a blue light blocking intervention in the evening and during nocturnal awakenings, in pregnant women. A low cost, safe treatment for sleep problems in pregnant women will have high public health interest, as available treatments are either hard to access or might carry risks. In the present study we investigated how two weeks use of BB-glasses affected sleep, subjectively and objectively, from pre- to posttreatment among women pregnant in the third trimester, compared to partially blue-blocking grey glasses. Also, symptoms of insomnia, evening sleepiness and evening activation were examined.

## Method

### Trial design

This study was a randomised double blind parallel group controlled trial, registered at ClinicalTrials.gov (NCT03114072), investigating an intervention to improve sleep in pregnant women in the third trimester.

The trial was conducted over three consecutive weeks, one baseline week followed by two intervention/control weeks.

### Participants

Healthy nulliparous women, about 24 gestation weeks, were recruited between May 2017 and April 2019, during their standard health control. Recruitment was mediated by consulting midwives at antenatal-healthcare centers in the Municipality of Bergen, Norway. All participants were provided information about the study (oral and written form) by the consulting midwife. If the pregnant women consented to receive more information or participate, further information was provided by the researcher (first author). Inclusion criteria were: 1) nulliparous women, 2) expecting one child, 3) being in the third trimester of a normal pregnancy (free from obstetrical complications), 4) able to wear an actigraph during daytime and nighttime for all three weeks and, 5) able to complete questionnaires in Norwegian. Exclusion criteria were: 1) somatic or psychiatric disorders, 2) fever and other health conditions affecting sleep, 3) working nights during the study protocol or 4) having a condition affecting the translucency of the eyes To be able to exclude women with serious eye-conditions affecting the translucency of the eyes, the red reflex [39] of both eyes was assessed. The participating pregnant women started the data collection between pregnancy week 27 to 32, mean week 29+0 days. For baseline data, the participants were assessed with subjective and objective measures for 7 days.

### Participant characteristics

Self-reported questions were administered to obtain information about maternal age, marital/partner status (married/cohabitating, single, separated/divorced, widow), level of education (high school and below, college and above), income (NOK<600 000, NOK >600 000; 10 NOK ≈ 1 US $), number of people living together in the household (partner, parents, parents in law, children, none, other), smoking (daily, less than daily, never), physical- and relaxing activity.

### Interventions

The interventions comprised of BB-glasses (Uvex Skyper S1933X, by Honeywell, Smithfield, RI, USA. www.uvex.us) blocking 99% of wavelengths shorter than 530 nm, and circa 15% of the light were in the remaining visual spectrum. The control condition were light grey glasses (Uvex Skyper S1905, by Honeywell, Smithfield, RI, USA. www.uvex.us) blocking approximately 50% of wavelengths shorter than 530 nm, and 30–50% of light were keeping in the remaining visual spectrum. A partial blue-blocking control group was used as this was assumed to maintain the placebo effect also in cases of knowledge of BB-glassesParticipants in both groups were instructed to wear the glasses from three hours before normal bedtime at night, and if needed, also when going to the bathroom etc. during the night until final awakening in the morning. They were instructed to report if they could not adhere to these instructions.

The participants were informed to contact the research team if they experienced any side-effects after start wearing the glasses, and were also probed for side-effects after study completion.

### Outcomes

The primary outcomes were total sleep time (TST), midpoint of sleep, sleep efficiency (SE) and daytime functioning, measured subjectively and objectively. The secondary outcomes were subjective symptoms of insomnia (Bergen Insomnia Scale; BIS), sleepiness prior to turning the lights off (Karolinska Sleepiness Scale; KSS), and evening activation (Pre-Sleep Arousal Scale; PSAS).

#### Subjective measure of sleep

*A sleep diary* was completed every morning. The sleep diary included items on number and duration of naps during the day, use of sleep medication (yes/no), bedtime, lights-out time, sleep latency, number of nocturnal awakenings, wake after sleep onset (WASO), waking and rise time. Included were also items assessing sleep quality and daytime sleepiness [40]. The outcomes used in the present study were total sleep time (TST)(min), sleep efficiency (SE%) (total sleep time/time in bed *100%), midpoint of sleep (TST:2)(hh:mm) and daytime functioning (score points).

*The Bergen Insomnia Scale (BIS)* was administered at study initiation and at day 21, the last day of participation. Originally the time frame was last month, but this was changed to last week in order to capture the rapid change in sleep that may occur during pregnancy and also to align the BIS with the time frame for the sleep diary and actigraphy measures. The BIS consists of six items. The first four pertain to sleep onset, maintenance, early morning wakening insomnia, and not feeling restored after sleep. The last two assess level of daytime impairment due to poor sleep and dissatisfaction with sleep. Each item is rated on a scale ranging from 0 to 7 days per week, providing a composite score point ranging from 0 to 42. Cut-offs of BIS indicating insomnia are scoring 3 or above on at least one of the first four items, and scoring of 3 or above on at least one of the last two items [41]. Cronbachs alpha for the BIS was .80 at study initiation and .78 at day 21.

*Karolinska Sleepiness Scale* (KSS) was completed every night before bedtime. The KSS was used to assess subjective sleepiness at two hours intervals from 8pm until sleep onset, measured latest at 2 am every night, and responses are provided on a 9-point Likert scale ranging from 1 (extremely alert) to 9 (extremely sleepy–fighting sleep) [42]. The mean score across four-time points (8pm, 10pm, midnight, 2am) in the evening/night was used.

*Pre-Sleep Arousal Scale (PSAS)* was completed every night before bedtime. The PSAS assesses psychophysiological arousal before sleep. The scale consists of 16 items and measures somatic (e.g. heart racing, shortness of breath, stomach upset) as well as cognitive (e.g. worry about falling asleep, depressing or anxious thoughts, mentally alert) components of arousal. Responses are recorded on a five-point Likert scale ranging from 1 (not at all) to 5 (extremely), providing a composite score ranging from 16 to 80. Higher scores indicate higher states of arousal [43]. Cronbachs alpha for the PSAS scale ranged across the 14 days between .65 (day 7) to .85 (day 2).

#### Objective measure of sleep

*Actigraphy*. To objectively estimate sleep patterns, each participant was asked to wear a commercially available wrist actigraph (Actiwatch Spectrum; Philips Respironics Inc.) on their non-dominant wrist, continuously throughout the study period. The actigraph registered movements by a piezoelectric accelerometer and epoch length was set to thirty seconds and the sensitivity was set to medium. The participants were instructed to press the event button on the actigraph to indicate when they turned off the light and tried to sleep, and when they finally woke up in the morning. Data were converted to objective sleep parameters through the Actiware software (version 6.0.9, Philips Respironics Inc.). Rest intervals were manually set based on visual determination of raw data, by the use of motor activity, light exposure, event-markers and also supported by sleep diary data. This approach was in line with international recommendations for the use of actigraphy data in sleep research [44]. Rest interval onset was set at marked decrease in activity (<50/min), event-marker followed by a sustained decrease in activity, or marked sustained decreases in light exposure (<8 lux). Rest interval termination was set at marked sustained increase in activity (>50/min), event- marker and/or sustained increases in light exposure (>8 lux). In cases where the event button was not pressed or where discrepancies between the sleep diary data and actigraph data were evident, duration of the sleep episode was set based on motor activity. Duration of the sleep episodes reflects time in bed minus sleep onset latency (SOL), time awake after sleep onset (WASO) and time in bed after final morning awakening [45]. Three sleep related outcome variables were derived: Total sleep time (TST)(min), sleep efficiency (SE)(%) and midpoint of sleep (hh:mm). Midpoint of sleep comprised a proxy of circadian phase [46].

### Sample size

The estimated sample size was based on effect sizes reported in previous studies that have used BB-glasses as treatment for sleep-disorders. These studies showed strong effects on sleep quality in one group of healthy individuals [32] and in persons diagnosed with ADHD [37]. Because sleep problems during late pregnancy also may be caused by various hormonal and mechanical factors, we expected a medium effect size (Cohens d = 0.50) for the BB-intervention. Setting the alpha to .05 (two-tailed), power to .80, correlation between repeated assessments to .50 revealed that a minimum of 34 participants in total were needed to detect statistically significant time (pre vs. post) x group (BB vs. control condition) interaction effects [47].

### Randomization and blinding

The included participants were randomized by www.randomizer.org to either intervention (BB-glasses) or control condition (grey glasses). A research assistant packed the glasses into an opaque brown paperbags. Based on unique numbers on the paper bags a randomization key not available to the researchers were made. Condition was first revealed to the first author when participants were handling in completed post-treatment questionnaires and the actigraph. All participants received the same oral and written information about the purpose of the study; testing glasses filtering different wavelengths of light on sleep and mood. They were instructed to refrain from researching the topic of light and sleep, and in case they needed to contact the research team not to describe their glasses. Participants with knowledge of BB glasses were not excluded, since both glasses eliminated some wavelengths shorter than 530 nm. This way we regarded that the placebo-effect were preserved also in the cases of some previous knowledge on effects of blue-filtering devices.

### Statistical methods

Characteristics of the study participants were presented as means and standard deviations or numbers and percentages as appropriate. Participant compliance of use of glasses were tested by independent two-sample t-test. Based on cut-off points of BIS, number and percentage of participants were calculated for insomnia, and a chi square test was used to examine the changes in insomnia (worse, unchanged, improved).

A descriptive analysis of the pattern of change were calculated with mean and standard deviations of subjective measured TST, midpoint of sleep and SE for each day of the second intervention week. To examine the effect of BB-glasses on the primary outcomes TST, SE, midpoint of sleep, daytime functioning and secondary outcomes KSS and PSAS and BIS, analysis of covariance (ANCOVA) were performed by including the baseline outcome measure as a covariate in regression models. The effect estimates were calculated as difference in means with 95% confidence intervals (95% CI) between BB-glasses and control glasses. The p-values for within group change in outcomes were calculated by paired t-test. Further, effect sizes (Cohens d) for both within and between groups were calculated. Analysis was conducted on per protocol set, hence excluded participants were not included in the analysis. Participants with mean TST <200 minutes per week were defined as outliers, and were excluded from the analysis.

All statistical analyses were performed using IBM SPSS Statistics version 25 (SPSS, Chicago, IL, USA), Stata IC version 16 (Stata Statistical Software, College Station, TX, USA) and R version 3.5.1 [48].

### Ethical considerations

The Regional Committee for Medical and Health Related Ethics, in Western Norway, approved the study (2016/1394/REK vest). All participants provided written informed consent before inclusion. After completed participation, all participants were debriefed about the aim of the study, and also offered BB-glasses as a compensation.

## Results

**Fig 1:**
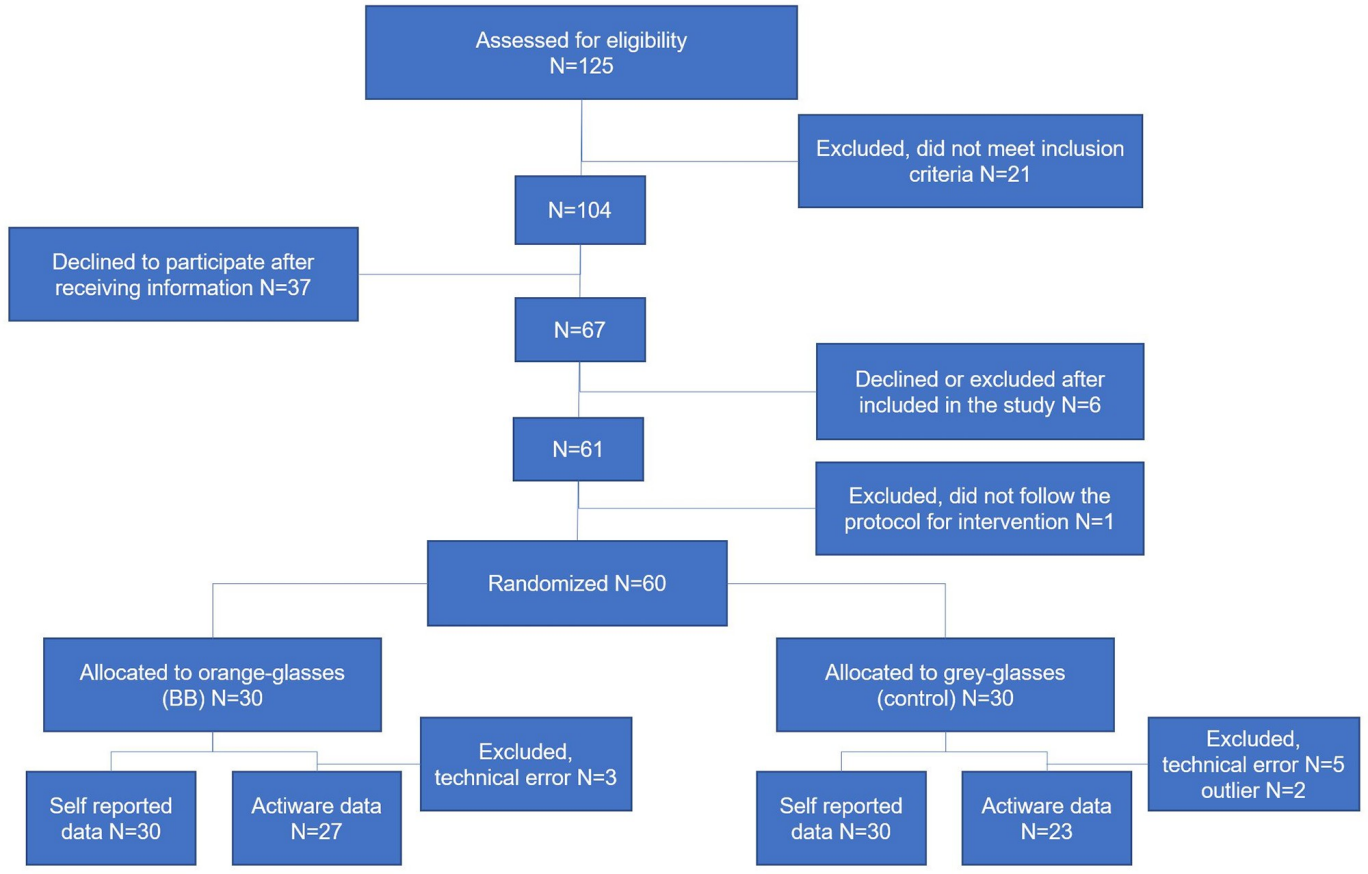
Flowchart of enrollment of pregnant women in the study.

Fig 1 presents a flowchart of enrollment. In total, 125 pregnant women were assessed for eligibility. After adherence to the inclusion and exclusion criteria and following elimination of those who refused to participate, a sample of 60 pregnant women were enrolled. The sample of pregnant women were evenly assigned to the two groups (BB group n = 30, control group n = 30). The reasons for exclusion of 7 participants were: preterm birth, fire in own home, severe malaise as a side-effect of the glasses, allergic reaction to the actigraph, not capacity to participate and discomfort wearing glasses. There were no missing data for the self-reported primary and secondary outcomes, except one missing follow-up value for the BIS. Actigraphy data from 8 women were excluded from the analyses because of technical errors, and data from further 2 were eliminated because of outliers. Accordingly, data from these individuals were not included in the regression analyses.

Participant characteristics for each group are presented in Table 1. The mean age for the intervention group was 30.0 (SD 3.7) years and 31.0 (SD 4.2) years for the control group; for the whole sample it was 30.5 (SD 4.0) years. In all, 96.7% of the primipara women were married or living with a partner, 83.3% had education at college level or above, 83.4% had an income of 600 000 NOK (≈ 60 000 US $) or more. Only one pregnant woman reported she was smoking. None of the pregnant women reported consumption of alcohol during the study weeks.

**Table 1:** Demographic factors for the blue-blocking- and control-group (self-reported data).

Characteristics	Total, both groups	Blue blocking group	Control group
N	60	30	30
**Age, mean (SD)**	30.5 (4.0)	30.0 (3.7)	31.0 (4.2)
**Marital status, N (%)**			
Married/ Cohabitating	58 (96.7)	30 (100%)	28 (93.3%)
Single	2 (3.3)	0	2 (6.7%)
**Education, N (%)**			
< = Senior high school	10 (16.7)	6 (20%)	4 (13.3%)
College and above	50 (83.3)	24 (80%)	26 (86.7%)
**Income, N (%)**			
< 600 000 NOK	10 (16.7)	5 (16.7%)	5 (16.7%)
>600 000 NOK	50 (83.4)	25 (83.3%)	25 (83.3%)
**Adult, total in household, N (%)**			
1	2 (3.3)	0	2 (6.7%)
2	57 (95.0)	29 (96.7%)	28 (93.3%)
4	1 (1.7)	1 (3.3%)	0
**Children, total in household, N (%)**			
0	58 (96.7)	30 (100%)	28 (93.3%)
1	1 (1.7)	0	1 (3.3%)
3	1 (1.7)	0	1 (3.3%)
**Smoking, N (%)**			
Daily	1 (1.7)	1 (3.3%)	0
Not at all	59 (98.3)	29 (96.7%)	30 (100%)
**Physical activity (min), mean (SD)**	23.8 (33.8)	29.7 (39.0)	18.0 (26.5)
**Relaxing activity (min), mean (SD)**	5.9 (18.0)	7.0 (19.5)	4.8 (16.4)
**Pregnancy week, mean (SD)**	29.1 (1.2)	28.9 (1.1)	29.3 (1.3)
**Participant compliance, use of glasses (min), mean (SD)** ^a^	169 (38.3)	173 (28.9)	165 (45.5)
**Insomnia (BIS), N (%)**			
**Baseline**	23 (38.3)	16 (53.3)	7 (23.3)
**Week 3**	29 (49.2)	14 (46.7)	15 (51.7)

Note. N = Number of participants; SD = standard deviation; NOK = Norwegian kroner; 10 NOK ≈ 1 United States dollar (US $); min = minutes.

^a^The difference were tested by unpaired t-test: t = .81, df = 58, p = .420.

Number of participants using the glasses less than instructed (180 min): ≤ 160 min = 23, ≤ 90 min = 23, 0 min = 11 (occurred once).

**Fig 2:**
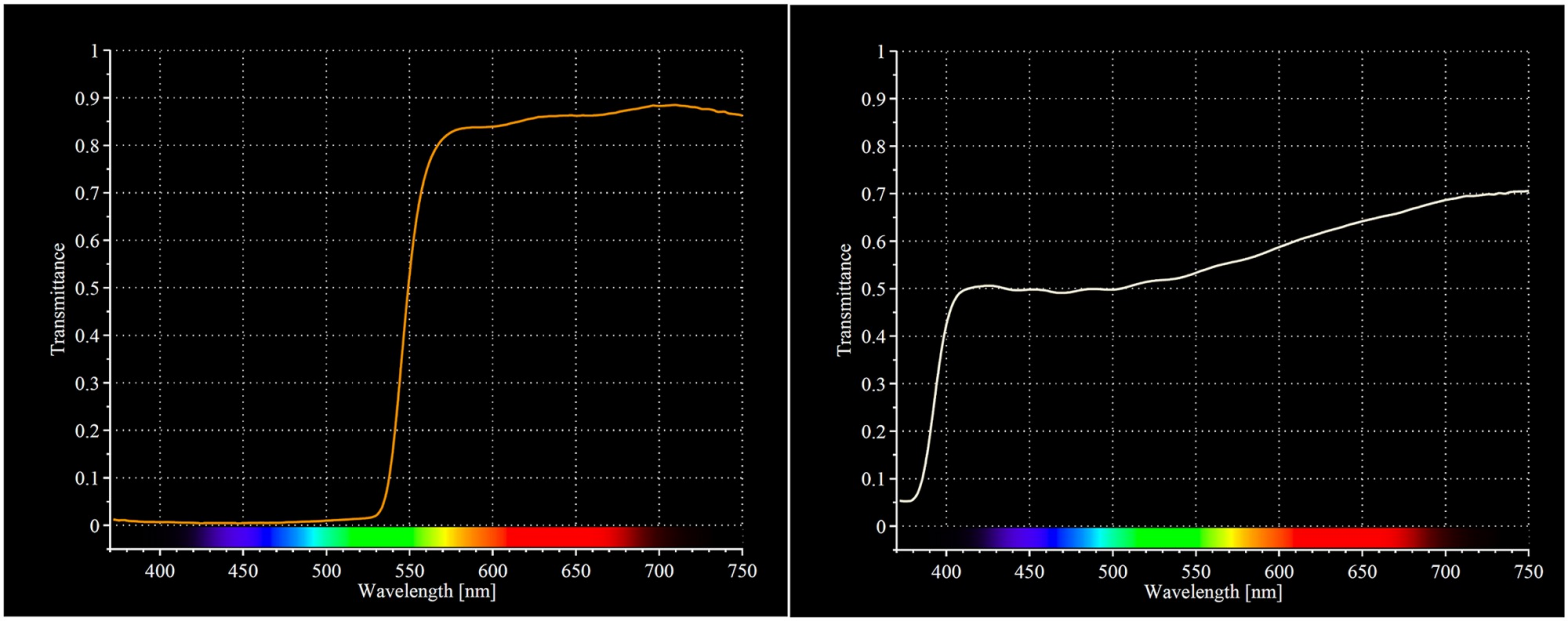
Irradiance spectra from intervention- and control glasses. Note the near complete filtering of blue light spectral irradiance (< 530 nm) of the BB-glasses.

During the second intervention week, the glasses were on average worn for 173 min per evening (SD 28.9) in the BB-group and for 165 min (SD 45.5) in the control group. This difference was not significant (t = .81, df = 58, p = .420). About one third of the sample (N = 23) reported they wore the glasses for a shorter time than instructed (range 0–160 min). For those reporting an event of non-adherence for an entire evening, this happened only once (N = 11). The irradiance spectra for the respective glasses are illustrated in Fig 2.

At baseline, in total 38.3% scored above cut-off for insomnia compared to 49.2% posttreatment. Within the BB-group 53.3% (baseline) and 46.7% (posttreatment) scored above cut-off compared to 23.3% (baseline) and 51.7% (posttreatment) within the control group. A change in insomnia diagnosis (worse, unhanged, improved) from baseline to posttreatment was investigated, showed in Supporting information (S1 Table). A total of 6 (20%) of the pregnant women in the BB-group improved, compared to 1 (3.3%) in the control group, and 4 (13.3%) in the BB-group compared to 9 (30%) in the control group worsen. A chi square test showed these changes were not significant (X^2^ = 5.5, df = 2, p = .064).

Only two participants reported they had some previous knowledge about the effect of BB glasses.

Table 2 displays the results of mean difference of outcome measure before and after intervention as well as from the ANCOVA analyses regarding the effects of BB-glasses and control glasses on the primary sleep outcomes variables and secondary outcomes KSS, PSAS and BIS. Within group change showed a significant decrease only for SE in actigraphy data in both groups, and an increase for the BIS score in the control group. The effect sizes were low for all outcome variables, both within and between groups. The BB-group showed for subjective TST an increase of 8 minutes and for control glasses 4 minutes. The corresponding actigraphy data showed a 5 minutes increase in the BB-group and a decrease of 35 minutes in control group. Regarding actigraphy data SE was above the suggested cut-off limit for baseline, but not at posttreatment. The control group lead to a decrease of SE from 85.5% (SD 5.5) to 79.0% (SD 16.6). The corresponding values for the BB-glass group were 85.6% (SD 5.6) and 84.9% (SD 5.7), respectively.

**Table 2:** Outcome at posttreatment.

Outcome	Blue blocking group		Control group		Effect size between groups ^a^	Estimated mean Difference (95%CI)^b^	P value
	N	Mean (SD)	N	Mean (SD)			
**Self-reported data**:							
**Total sleep time (min)**							
Baseline	30	439.0 (38.2)	30	450.0 (54.2)			
Week 3	30	447.7 (55.7)	30	454.5 (45.5)	-.134	.78 (-19.7, 21.3)	.939
P value within groups		.314		.505			
Effect size^c^		.175		.087			
**Midpoint of sleep (hh:mm)**							
Baseline	30	03:55 (00:42)	30	03:51 (00:46)			
Week 3	30	03:47 (00:45)	30	03:52 (00:50)	.116	-8.9 (-23.7, 5.9)	.234
P value within groups		.196		.786			
Effect size^c^		.192		-.023			
**Sleep efficiency (%)**							
Baseline	30	85.6 (11.1)	30	85.8 (10.3)			
Week 3	30	86.7 (6.0)	30	87.1 (5.9)	-.067	-.06 (-1.9, 1.8)	.948
P value within groups		.070		.099			
Effect size^c^		.193		.216			
**Daytime functioning (score points)**							
Baseline	30	3.2 (.6)	30	3.3 (.7)			
Week 3	30	3.3 (0.7)	30	3.5 (.6)	-.307	-.05 (-.33, .22)	.703
P value within groups		.119		.198			
Effect size^c^		.152		.304			
**Bergen Insomnia Scale (score points)**							
Baseline	30	13.4 (8.0)	29	8.9 (6.5)			
Week 3	30	11.8 (6.8)	29	11.2 (7.8)	.082	-2.4 (-5.6, .72)	.128
P value within groups		.176		.034			
Effect size^c^		.213		-.359			
**Karolinska Sleepiness Scale (score points)**							
Baseline	30	5.9 (.8)	30	5.8 (.9)			
Week 3	30	5.9 (1.1)	30	5.6 (1.1)	-.273	.25 (-.15, .66)	.218
P value within groups		.751		.141			
Effect size^c^		< .001		.193			
**Presleep Arousal Scale (score points)**							
Baseline	30	21.9 (3.2)	30	20.7 (4.0)			
Week 3	30	21.3 (3.6)	30	20.4 (3.5)	-.253	.05 (-.95, 1.0)	.922
P value within groups		.100		.346			
Effect size^c^		.173		.078			
**Actigraph data**:							
**Total sleep time (min)**							
Baseline	27	440.8 (43.0)	23	450.9 (39.1)			
Week 3	27	445.2 (53.4)	22	415.2 (93.2)	.115	13.0 (-9.5,35.5)	.251
P value within groups		.604		.137			
Effect size^c^		.089		-.302			
**Midpoint of sleep (hh:mm)**							
Baseline	27	04:26 (00:25)	23	04:27 (00:32)			
Week 3	27	04:28 (00:35)	22	04:18 (00:50)	-.010	2.1 (-11.6, 15.8)	.754
P value within groups		.694		.913			
Effect size^c^		-.061		.014			
**Sleep efficiency (%)**							
Baseline	27	85.6 (5.6)	23	85.5 (5.5)			
Week 3	27	84.9 (5.7)	22	79.0 (16.6)	.244	1.7 (-.4, 3.7)	.115
P value within groups		.049		.033			
Effect size^c^		-.124		-.342			

Note: N = Number of participants; SD = standard deviation; CI = confidence interval; min = minutes; hh:mm = hours and minutes; % = percent.

^a^Estimated with Cohens d, negative effect size indicating a negative trend at post value, or the control group are doing better than the blue blocking group.

^b^ Estimated by using analysis of covariance (ANCOVA) by including the baseline outcome measure as a covariate in linear regression models.

^c^Estimated with Cohens d, by paired t-test for within group change.

**Fig 3:**
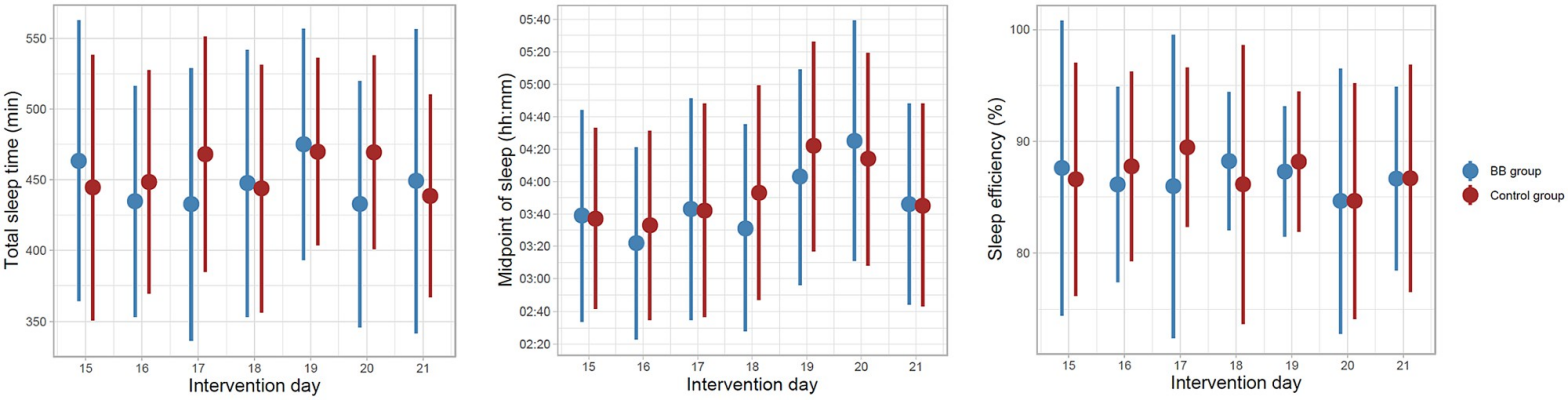
Daily sleep during the second intervention period (subjective data). The pattern of daily changes in total sleep time, midpoint of sleep and sleep efficiency presented with mean and standard deviation for the second intervention week.

The analyses for self-reported data for primary sleep outcomes showed no statistically significant difference between the groups at posttreatment in terms of TST (difference .78 [min], 95%CI = -19.7, 21,3), midpoint of sleep (difference -8.9 [min], 95%CI = -23.7, 5.9), SE (difference -.06 [%], 95%CI = -1.9, 1.8) and daytime functioning (difference -.05 [score points], 95%CI = -.33, .22) after using the baseline outcome measure as a covariate in regression models. Likewise, regression analyses showed no group difference in actigraphy data for the primary sleep outcomes TST (difference 13.0 [min], 95%CI = -9.5, 35.5), midpoint of sleep (difference 2.1 [min], 95%CI = -11.6, 15.8) and SE (difference 1.7 [%], 95%CI = -.4, 3.7) or for the secondary outcome measures BIS (difference -2.4 [score points], 95%CI = -5.6, .72), KSS (difference .25 [score points], 95%CI = -.15, .66) and PSAS (difference .05 [score points], 95%CI = -.95, 1.0). The pattern of daily change in subjective sleep through the last intervention week are presented in Fig 3, and shows a similar pattern, with some variation throughout the week.

There were some side-effects reported from use of both the intervention and control glasses. Reported side-effects of BB-glasses were: malaise (n = 2) restored after about 5 minutes, and headache, anxiety and depressive mood (n = 1) which lasted for 1.5 hour the first evening and 30 minutes the second evening while still wearing the glasses, and absent the third night. Side-effects reported by the control group comprised severe malaise (n = 1) in such a way that exclusion was necessary; headache (n = 1) the first evening, then restored; experienced watching double text/subtitles on the TV some evenings (n = 1).

## Discussion

The aim of the present study was to assess the effect of BB-glasses compared to partially blue-blocking grey glasses on sleep outcomes among nulliparous women in the third trimester of the pregnancy as such intervention previously never has been investigated in this population.

In terms of sleep, none of the sleep variables TST, SE, midpoint of sleep and daytime functioning showed any differential effects of BB-glasses and control glasses, neither when assessed with sleep diary nor by actigraphy. According to the score on the BIS, the KSS and the PSAS the BB-glasses did not show any statistically significant effect between the groups. The results suggested that compliance was high in both groups. Hence, lack of differential effects between conditions can probably not be attribute to lack of compliance.

Although a decrease in insomnia score for the BB-group and an increase for the control group posttreatment were shown on the BIS this was not statistically significant, even the control group improved significant within group. The change in the categorized insomnia diagnosis (worse, unchanged, improved) showed a trend toward improvement of insomnia status in the BB-group from baseline to posttreatment, whereas the opposite was the case for the control group, though not significant. At baseline the sample showed a mean total sleep time of approximately 7.5 hours based on sleep diary and actigraphy data, which were quite comparable to recommendations for total sleep time for women in the relevant age group [49]. Also, the pregnant women part taking in the intervention slept longer during baseline than a comparative group of non-pregnant women according to actigraphy data. In the weekend they tended to extend their sleep more than the non-pregnant group, hence they seemed to accumulate a sleep deficit during the week [29]. This indicate that this group of pregnant women slept overall quite well, and followed a sleep pattern which is common in working populations [50].

In the present study from pre to post-treatment the BB-group showed a small increase on TST both assessed with sleep diary and actigraphy, whereas this was only the case for TST assessed with sleep diary for the control group. Still the difference between the groups were statistically non-significant. Related to previous studies of healthy pregnant women, we would expect worsening of sleep in the third trimester compared to the two first trimesters [2–4]. This may have occurred in our sample but the women were not recruited before they were in the third trimester. However, other studies have shown a shorter average TST (range 6h 26 min-7h 19 min) [2, 3, 6, 51, 52] in the third trimester, than the present study (range 7h 25–34 min), except of the control group for actigraph data (6h 55 min). It is worth noting that the discrepancies between some of these studies and the present one in terms of TST were small (9–18 min), although the prevalence of insomnia were higher in other studies than the present [6, 52].

This study showed an earlier mean midpoint of sleep assessed with sleep diary in the BB-group and in actigraphy data for the control group, though not significant. The mean SE assessed with sleep diary was above the suggested cut-off of 85%, used as an indicator of adequate sleep quality, both at baseline and post-treatment for the BB-group and the control group. In regard of the actigraphy data SE was above the suggested cut-off at baseline, but not at posttreatment for both groups. Within group analyzes did show significance decrease in SE for both groups, although between groups neither of these findings were statistically significant. Previous studies show lower SE than our study [4]. Another study of pregnant women in Norway reported a lower SE but a small discrepancy in TST (9–18 minutes) compared to the present results [6, 52]. A possible explanation could be that our sample had shorter time in bed which typically result in higher SE. An SE above 85% indicates that this sample of pregnant women had good sleep quality. The reduction (not statistically significant) of SE in actigraphy data may reflect a decrease of sleep quality because of a further advanced pregnancy.

It could be argued that we could not expect the pregnant women in this study to improve sleep due to the intervention, as their baseline sleep overall was quite good. Still, pregnancy normally causes several physical and psychological changes, including poor sleep. Sleep deteriorates especially during the third trimester, characterized by longer sleep latency, decreased SE, longer WASO [4] and decreased TST [3] at night, compared to the two first trimesters. In previous research using BB-glasses in people with insomnia, TST has shown to be increased following use [35, 53]. It is well known that light exposure in the evening, can cause suppressed secretion of melatonin [30, 54], circadian disturbance and increased alertness [55, 56], even though some studies also report no alerting effects [15]. By blocking blue wavelengths from reaching the retina, BB-glasses prevent these such negative effects [18, 25]. Use of blue-blocking glasses (BB-glasses) in the evening allows the melatonin-production to follow the natural cycle of light and darkness, even when electric light and light-sources such as smart-phones, tablet, computers and TV are used [18]. In this regard it should be noted that the sample in the present study belongs to a group that usually uses electronic devices, also in the evening, even in bed [16, 33].

The mean score of daytime functioning and mean score of the KSS was within normal levels, which means they were neither sleepy in daytime nor alert during evenings. In addition, the low scores on the PSAS indicated low arousal in the evening right before bedtime. However, several aspects of pregnancy may increase stress, which may negatively affect sleep, especially if occurring close to bedtime. Women with stress-related sleep disturbances during pregnancy are more likely to experience insomnia [10]. In addition, pregnant women are known to be more sensitive to stressors, and elevated mood and depressive symptoms are common in pregnancy [6]. Mood changes are often associated with bidirectional alterations in sleep. It has further been shown that individuals with mood disorders seem to have elevated sensitivity to light [32]. Against this backdrop, we expected, despite relatively good sleep at baseline, that the BB-glasses would prevent negative development of sleep or even would improve sleep during the third trimester. Except for sleep efficiency, both groups did show a small improvement in sleep outcomes from pre- to posttreatment, although the between group changes were non-significant.

However, the present study cannot serve as basis for a general recommendation of BB-glasses as a sleep aid to pregnant women in general. Clinically there was a tendency for the women in the BB-group to fare better than those in the control group from baseline to posttreatment in terms of insomnia status. We suggest that effects of BB-glasses should be investigated in pregnant women with more sleep problems than in the present sample. Further, future trials should also include a control condition with no blocking of blue light.

The observed side effects (malaise, headache, lowered mood and anxiety) were transient, and equally represented in both groups (n = 3) except for severe malaise reported by one participant in the control-group causing drop-out. The frequency of side-effects is similar to that reported by Henriksen [57], while other studies have reported no adverse effects [32]. Our findings is in line with the previous conclusions from the literature that BB-glasses is a safe intervention when used in the evening and night [34, 38, 57]. It is of note that the side effects in five of the six cases disappeared during continued use, a notion that is of practical clinical value.

### Limitations and strength

The sample in this study reported good sleep overall, with 53.3% in the BB group and 23.3% in the control group fulfilling the criteria of insomnia at baseline. Thus, it is reasonable to assume that the presents sample may not be representative of pregnant women in general, and therefore limits the generalizability and the target validity of the present findings. Target validity might be limited in the present study [58], as only 38.3% of total sample initially fulfilled the criteria for insomnia. Another limitation of the present study concerns the relatively low number of subjects, making the study susceptible to type II errors. Still, a priori power analysis was calculated based on a previous study of sleep quality [32], and also based on persons diagnosed with ADHD [37]. Even so, several studies with a lower number of participants have shown positive effects of BB-glasses on sleep outcomes [32, 33, 35–37], which may be due to chance, although significant effects were reported. A common factor for these studies however was that the subjects reported strong symptoms of sleep-problems [38]. Also, replication based on a larger sample and with subjects fulfilling criteria for insomnia should also be conducted.

The sample of pregnant women in this study seems healthier than reported in previous studies of pregnant women in terms of sleep [1, 6]. It should also be noted that a vast majority of the participants were married or cohabitating, as well as had higher education and income than the general population, which might is a protective factor for poor sleep the link between insomnia and low socioeconomic status is demonstrated [59].

The present study is partly based on self-report data which may lead to recall bias [60], social desirability bias [61] and some common method bias [62]. Still, the randomization processes would prevent such biases to influence the results. Although actigraphy has shown low specificity in many studies [63], it has still shown to be sufficiently sensitive to detect changes in sleep duration in several studies [44]. A strength of the present study is the combination of subjective and objective sleep assessment, and an observation of a full week with baseline data and treatment effect data.

The control glasses used in the present study blocked 50% of blue wavelengths, which may also have provided some effect on the human melanopsin system. In order to take measures to counteract personal knowledge about blue light-filtering glasses among study participants, we considered that the control glasses should also have some blue-blocking filtering effect, and this way strengthen blinding of the participants. Two participants reported they had some previous knowledge about the effect of BB glasses. Future studies on effects of BB-glasses should avoid or keep to a minimum the reduction of melanopic lux yielded by the control condition.

## Conclusions

In this study of 60 healthy Norwegian pregnant women in the beginning of the third trimester the participants had overall well-preserved sleep. The use of BB-glasses compared to grey partially blue-blocking glasses did not show a statistically significant difference in sleep outcomes between the groups, neither assessed by self-report or actigraphy. Side effects were low-frequent and mostly transient.

## Supporting information

S1 Table Changes in insomnia diagnosis from baseline to posttreatment (self-reported data). (DOCX)

S1 Checklist CONSORT 2010 checklist of information to include when reporting a randomised trial*. (DOC)

S1 Protocol Nightly light exposure in pregnancy: Blue-blocking glasses as an intervention to ease sleep disturbances and to improve mood. (DOCX)

S1 Dataset Table 1, supporting table and Fig 3. (XLSX)

S2 Dataset Table 2. (XLSX)
