# Supplementary material for: A randomized controlled trial on the effects of blue-blocking glasses compared to partial blue-blockers on sleep outcomes in the third trimester of pregnancy
Source: PLoS One. 2022 Jan 28;17(1):e0262799. doi: 10.1371/journal.pone.0262799 (PMC8797219; doi:10.1371/journal.pone.0262799)
Supplement: S1 Table — (DOCX) [file pone.0262799.s001.docx]

**S1 Table. Changes in insomnia diagnosis** **from baseline to posttreatment (self-reported data)***.*

|  | **Total, both groups** | **Blue-blocking group** | **Control group** |
| --- | --- | --- | --- |
| N | 60 | 30 | 30 |
| **Insomnia change (BIS), N (%)** |  |  |  |
| Worse | 13 (21.7) | 4 (13.3) | 9 (30) |
| Unchanged | 40 (66.7) | 20 (66.7) | 20 (66,7) |
| Improved | 7 (11.7) | 6 (20) | 1 (3.3) |

N=Number of participants; BIS = Bergen Insomnia Scale.

Chi square test of changes in insomnia diagnosis from baseline at week 1 to posttreatment at week 3; X^2^=5.5, df=2, p=.064.
