# Supplementary material for: A randomized controlled trial on the effects of blue-blocking glasses compared to partial blue-blockers on sleep outcomes in the third trimester of pregnancy
Source: PLoS One. 2022 Jan 28;17(1):e0262799. doi: 10.1371/journal.pone.0262799 (PMC8797219; doi:10.1371/journal.pone.0262799)
Supplement: S1 Protocol — (DOCX) [file pone.0262799.s003.docx]

**Nightly light exposure in pregnancy: blue-blocking glasses as an intervention to ease sleep disturbances and to improve mood**

This project will contribute with new knowledge on how nightly light exposure in pregnant women in their third trimester affect their sleep. Importantly, the project initiates new research on non-pharmacological treatment option of sleep disturbances by blocking the blue wavelengths of light in the evening and during nocturnal awakenings. Blue light is known to increase alertness through a recently described retinal receptor specialized for detecting daytime light signals. This project is highly innovative and may have significant practical implications

**Background**

Sleep disturbances are common in pregnancy (1-3), and the incidence increases during the third trimester. Poor sleep quality is reported in 39% of women in the early pregnancy, and in 54% during the third trimester (1). Up to 97% of pregnant women report nocturnal awakenings (4). For some, the sleep problems continue postpartum (2).

There are many conditions and sleep disorders that can cause disrupted sleep among pregnant women. Sleep apnea (breathing cessations during sleep), restless legs syndrome (an irresistible urge to move legs), and insomnia (difficulty in falling asleep and/or maintaining sleep) are common (5). Insomnia has been reported by so many as 62% of pregnant women, a number that is significantly higher than that found in the general population (10-15%) (6). The various hormonal and mechanical influences that contributes to fragmented sleep during pregnancy also reduce the time spent in the deep sleep (1). Disrupted sleep among pregnant women also include nocturia (a frequent need to get up and urinate at night), dyspnea (shortness of breath), nasal congestion, muscular aches and pelvic pains, fetal activity, leg cramps and also reflux (5).

Sleep is restorative for the brain, immune system, metabolic system, hormone regulation and many more physiological functions. Sleep disturbances are generally associated with a wide range of physical health problems, and impaired mental health (7). Insomnia typically co-occurs with several mental disorders, especially anxiety and depression. Cross-sectional and longitudinal studies have suggested that insomnia is a signiﬁcant risk factor for the development of depression (8, 9) and anxiety (10, 11). Depressive symptoms have been reported with 14.6% of pregnant women and were strongly associated with insomnia in the third trimester of pregnancy (6). Several studies have shown an association between sleep disturbances in pregnancy and adverse pregnancy outcomes for both mother and fetus, including preeclampsia, elevated serum glucose, depression, prolonged labor, cesarean birth, alteration in fetal growth and preterm birth (5, 12, 13). Also, long-term health outcomes such as hypertension, diabetes and obesity are demonstrated (12-14)*.*

Effective and safe treatments for sleep disturbances during pregnancy are current lacking. Medical treatments with medications such as benzodiazepines, hypnotic benzodiazepine receptor agonists, antidepressants, melatonin and antihistamines are available, however there is little research on the effects and potential side-effects, especially for the fetus, when used during pregnancy (15). Hence, medication is not a recommended first-line treatment (5). Non-pharmaceutical options, such as sleep hygiene counselling or cognitive behavior therapy are safe options, but evidence for a clinically effect on the pregnant population is scarce (5, 15). Another limitation is that treatments such as cognitive behavior therapy are time-consuming, costly, and often not readily available.

The nocturnal awakenings often involve the pregnant women to leave the bed and consequently light exposure. The electric light in the evening and during night increase alertness, disturb sleep, shift the timing of the circadian clock and impair the brains’ restorative slow waves during deep sleep (16). Sleep, alertness and circadian rhythms are biological processes that display endogenous, entrainable oscillations of about 24 hour. According to the phase response curve for light, exposure of light in the evening and night typically delays the circadian rhythm (17). Light also enhances alertness leading to a wakeful state (18). Hence, getting up several times a night during pregnancy may produce an imbalance between the internal biological timing, the sleep-wake schedule and quality and quantity of sleep.

**Blue blocking glasses as a safe intervention**

Recent studies have shown that use of blue-blocking glasses (BB-glasses) in the evening improve sleep quality (subjectively reported) among persons with insomnia, and prevent alertness caused by blue-light emitting screens such as smartphones and iPad (19, 20). The mechanisms behind involves specialized retina ganglion-cells (intrinsically photoresponsive Retinal Ganglion-Cells, IpRGC), responsible for detecting and conveying the circadian day light signal directly to the SCN. IpRGC respond to a narrow band of wavelengths, most sensitive to the frequencies between 446 and 484 nm; the blue light (21, 22). Amber/orange tinted glasses works as a filter by blocking wavelengths shorter than 530 nm and have been shown to preserve normal nocturnal melatonin levels in a light environment that otherwise completely suppresses the natural production of melatonin (23). “Virtual darkness” to the SCN is therefore achievable by blocking the blue wavelengths of light (24, 25). The use of BB-glasses in evening/night allows the melatonin-production to follow the natural cycle of light and darkness, even when electric light and light-sources such as smart-phones, iPad and TV are used. In a recent case study, we have demonstrated that sleep is rapidly transformed from very irregular to markedly regular during the use of BB-glasses (26). Importantly, blocking blue light during the evening and night is not reported to produce any harmful side-effects, and in contrast to medication it does not depend on detoxification processes in the liver. Treatment studies show that use of BB-glasses are effective in improving mood, as those wearing BB-glasses recovered more quickly for postpartum depression (19).

Women’s health in reproductive age is a deciding factor for the future population (27). Total sleep time and quality of sleep play an important role in both the mothers and the child’s health (5, 28). New knowledge about preventing sleep problems in pregnancy may avoid several of the aforementioned negative health outcomes (12, 13) Clinical trials of safe interventions for women with sleep disturbances during pregnancy are thus strongly warranted (5). The existing studies suggest that use of the BB-glasses may improve sleep quality by influencing the sleep physiology in different populations. Based on this, we want to examine the effects of BB-glasses to prevent ill-timed light exposure at night in healthy pregnant women during the third trimester from causing disturbed sleep. To our knowledge, this will be the first clinical trial using an optical filter as intervention for sleep problems in pregnant women, and the project has thus both high clinical and scientific importance.

**Aims**

The main aim of this project is to investigate the effect of BB-glasses used in the evening and night on Dim Light Melatonin Onset (DLMO), alertness (subjectively), sleep quality (subjectively and objectively), positive and negative affect and mood for pregnant women in the third trimester. Furthermore, we want to establish the prevalence of sleep disturbance, sleep-pattern and sleep-related behavior in the study population, and the association with daily/nightly light exposure.

The sub goals are:

1. To assess the light environment and sleep-pattern among pregnant women in the beginning of the third trimester.
2. To investigate the effect of blue-blocking glasses on sleep quality among pregnant women in the beginning of the third trimester.
3. To investigate the effect of blue-blocking glasses, in the evening and during the night, on DLMO, positive and negative affect and symptoms of anxiety and depression among pregnant women in the beginning of the third trimester.

**Hypotheses**

1) Exposure to light in evening and night among pregnant women are positively associated with evening and nocturnal alertness, delayed DLMO and inversely related to sleep quality.

2) Use of blue-blockers in the evening and night advances DLMO, facilitates sleep (subjectively and objectively) and decreases daytime sleepiness in pregnant women in the third trimester.

3) Use of blue-blockers in the evening and night lead to an increase in positive affect and decrease in negative affect, improved mental health as indicated by improved mood, reduced symptoms of depression and anxiety in pregnant women in the third trimester.

**Methods**

**Design**


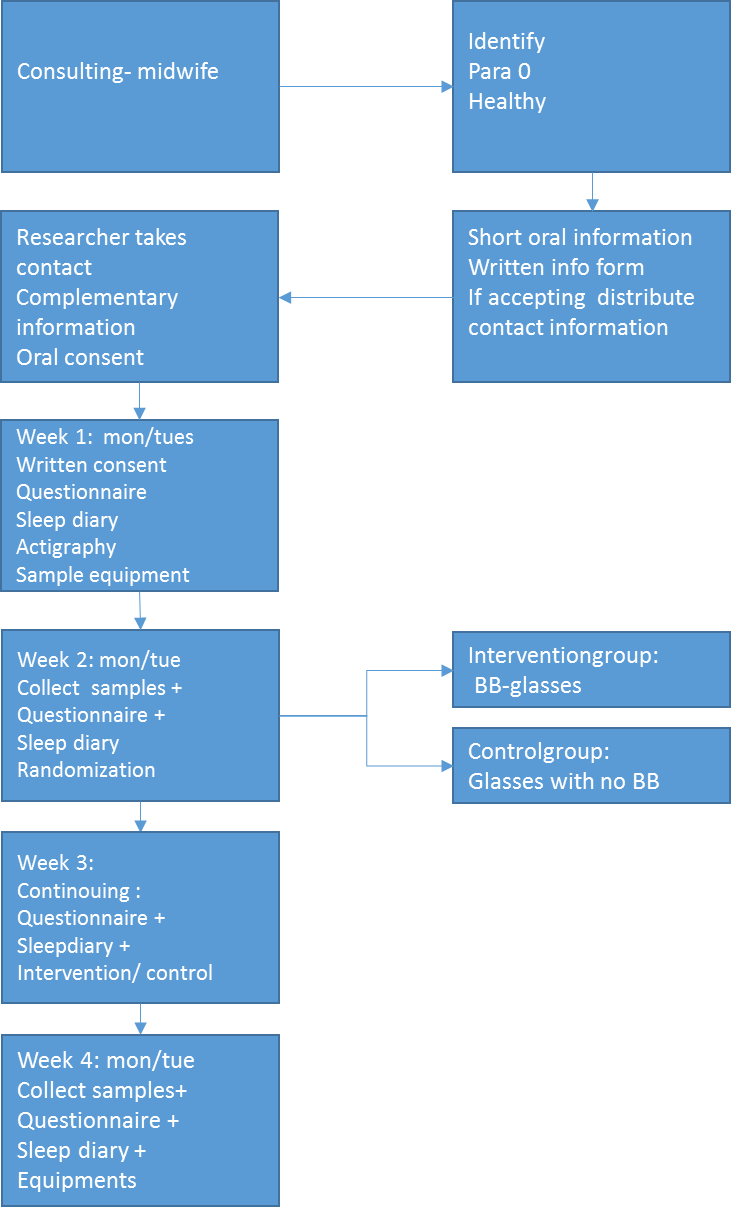
This is a prospective longitudinal randomized controlled trial.

**Study population and procedure**

A sample of 60 pregnant women will, during the standard control about gestational week 24, be recruited through consulting midwifes at antenatal-healthcare centers in the Municipality of Bergen. We will recruit until 60 participants have completed the protocol.

The inclusion criteria are; 1) healthy nulliparous women, 2) expecting one child, 3) being in the third trimester of a normal pregnancy, 4) not working at night during the study protocol, 5) able to wear an actigraph during the daytime and nighttime.

Exclusion criteria are; fever and other health conditions affecting sleep.

All participants will be given information about the study (oral and written form) by the consulting midwife. If the pregnant women consent to participate in the study, further information is provided by the research team. To exclude women with serious eye-condition affecting the translucency of the eyes, the red reflex of both eyes will be checked. The pregnant women will start going through the study protocol at gestational week 28. For baseline data, the participants will be assessed with subjective and objective measures for 7 days followed by 14 days of the BB-intervention/control-intervention. See Table 1. Consenting participants will be randomized (by www.randomizer.org) to either intervention/control condition.

Figure 1 Flow diagram of recruiting and data collection progress

|  | **Week 1 Day 1** | **Day 2–6** | **Day 7** |
| --- | --- | --- | --- |
| Morning  During the day  Evening | Actigraphy/light recorder  Sleep diary, incl. items about restless legs (RLS) , alcohol and physical activity  Demography  The Relationship Satisfaction Scale (RS)  Bergen Insomnia Scale (BIS)  The Epworth Sleepiness Scale (ESS)  The Beck Anxiety Inventory (BAI)  The Beck Depression Inventory (BDI-II)  Perceived Stress Scale (PSS)  The Penn State Questionnaire (PSWQ)  Karolinska Sleepiness Scale (KSS)  Positive and Negative Affect Schedule (PANAS)  Preesleep Arousal Scale (PSAS) | Actigraphy/light recorder  Sleep diary  PANAS  KSS  PSAS | Actigraphy/light recorder  Sleep diary  PANAS  KSS  PSAS  Melatonin sample from saliva |
|  | **Week 2 Day 8** | **Day 9-14** |  |
| Morning  During the day  Evening | Actigraphy/light recorder  Sleep diary  ESS  (PSS)  (PSWQ)  PANAS  KSS  PSAS  *Randomization: BB-glasses/control-glasses* | Actigraphy/light recorder  Sleep diary  PANAS  KSS  PSAS  *BB-glasses/control-glasses* |  |
|  | **Week 3 Day 15** | **Day 16-20** | **Day 21** |
| Morning  During the day  Evening | Actigraphy/light recorder  Sleep diary  ESS  PANAS  KSS  PSAS  *BB-glasses/control-glasses* | Actigraphy/light recorder  Sleep diary  PANAS  KSS  PSAS  *BB-glasses/control-glasses* | Actigraphy/light recorder  Sleep diary  ESS  BIS  PANAS  KSS  PSAS  BAI  BDI-II  PSS  PSWQ  Melatonin sample from saliva  Ending intervention; *BB-glasses/control-glasses* |

Table 1 Instruments and procedures

**Intervention**

The intervention comprises blue-blocking glasses (orange-tinted), which remove more than 90% of the blue wavelengths; wavelengths shorter than 530 nm. (26) The control-glasses will be light grey, with no blue-blocking effect.

The intervention group and control group will be instructed to wear the BB-glasses/control glasses from two hours before normal bedtime at night, until they go to bed. They will be informed to not expose themselves for light without wearing the BB-glasses during the night, and if needed, like going to the bathroom during night, to wear the BB glasses.

**Instruments/Measures**

*Actigraphy* (29). For indirect measure of sleep patterns, we will use a commercially available actigraph (Actiwatch Spectrum from Philips Respironics). This actigraph looks like a watch and is constructed to register motor activity. It is worn around the wrist and registers movements by a piezo-electric accelerometer. Motor activity for periods of thirty seconds will be recorded. Data can be converted to objective sleep parameters. Actigraphy has shown to be sufficiently sensitive to detect changes in sleep length (30).

*Light recorder* (29). The actigraph Actiwatch is also constructed to register light; total white light, blue, green and red light.

*Melatonin samples* from saliva for establishing DLMO on day 7 during baseline-week, and repeated the last evening of the consecutive intervention-week day 21.

*Sleep diary* (31). Daily subjective estimates of bedtime, rise-time, sleep latency, number of awakenings, wake time after sleep onset, final awakening time, rise time, total sleep time, sleep efficiency, sleep quality and daytime functioning.

*Pregnancy related complaints* during the night will be daily measured by self-reporting of itching of the skin, leg cramps, restless legs, pelvic pain, back pain, fetal movements, nausea, vomiting, reflux and nocturia. The response scale range from 1 (no complains) to 5 (extremely).

*Restless Legs* (RLS) (32) will be assessed using the diagnostic criteria from the International Classification of Sleep Disorders – third edition.

*Physical activity* (33) will be daily measured by asking “How many minutes were you physical active, which was enough to raise your breathing rate?” Participants will be asked to fill in the amount of minutes every day.

*Alcohol consumption* will be measured as daily subjective estimates by asking “Did you drink alcohol yesterday, and how many alcoholic drinks?” by responding 0 for no alcohol, or specify the number of alcoholic drinks.

*The Bergen Insomnia Scale (BIS)*(34). The BIS assesses symptoms of insomnia during the previous week, and are related to sleep onset, maintenance, early morning wakening insomnia, not feeling adequately rested, experiencing daytime impairment and being dissatisfied with current sleep. The response alternatives range from 0 (no days per week) to 7 (all days per week).

*Karolinska Sleepiness Scale* (KSS) (35)**.** Subjective sleepiness just prior to turning the lights off will be assessed with the KSS. KSS assesses subjective state sleepiness and consists of one item with a scale ranging from 1 (= being very alert) to 9 (= the highest level of sleepiness).

*Positive and Negative Affect Schedule (PANAS)* (36)***.*** Changes in positive and negative affect and emotions will be measured by this 20-item self-report schedule. This is a five-point response scale ranging from 1 (very slightly) to 5 (extremely). The participants will be asked to complete PANAS just prior to turning the lights off.

*Pre-Sleep Arousal Scale (PSAS)* (37) consists of 16 items and measures eight cognitive (e.g., intrusive thoughts) and eight somatic (e.g., sweating) manifestations of arousal during before sleep onset. This is a five-point response scale ranging from 1 (not at all) to 5 (extremely). Higher sum scores of the 16 items indicate higher state of arousal.

*The Epworth Sleepiness Scale* (ESS) (38) is a trait measure of daytime sleepiness. Respondents are instructed to rate off in eight different situations on a scale ranging from 0 (would never doze off) to 3 (a high risk of dozing).

*The Beck Anxiety Inventory (BAI)* (39) is a 21-items list of symptoms of anxiety for self-report questionnaire for measure the severity of anxiety. The instrument has a four-point response scale ranging from 0 (not at all) to 3 (Severely – it bothered me a lot). Higher sum scores of the 21 items indicate higher state of anxiety.

*The Beck Depression Inventory (BDI-II)* (40) consisting of 21 groups of statements measuring the experience of depression during the last week. This is a four-point scale, ranging from 0 (e.g. I do not feel sad) to 3 (e.g. I am so sad or unhappy that I can’t stand it). Higher sum scores of the 21 items indicate higher state of depression.

*The Penn State Worry Questionnaire (PSWQ)* (41) measures the trait of worry by 16 items. The response scale range from 1 (not typical) to 5 (very typical) and indicate the state of worry.

*Perceived Stress Scale (PSS)* (42). The PSS measures the degree to which situations in one's life are appraised as stressful. The instrument consist of 14 items, each scored on a five-point scale ranging from 0 (never) to 5 (very often).

*Relationship Satisfaction Scale (RSS)(43).* This instrument measures marital satisfaction and relationship quality. It consists of a 10-items list of statements related to satisfaction with the relationship, and has a 6-point scoring scale ranging from 1 (very agree) to 6 (strongly disagree). This measure is included in order to describe the characteristics of the study sample and as a control variable.

*Sociodemographic factors* will be measured by self-reports in order to describe the characteristics of the study sample. The participants will be asked about:

*Marital status;* by selecting one of the following categories: ‘married’, ‘cohabiting’; ‘single’ and ‘separated/divorced’.

*Level of education;* by indicating the completed level of education in 9 categories. The categories are divided on six groups based on number of in years of education. The responses will be scored from 1 (lowest education level) to 6 (highest education level).

*Income* by asking: ‘What is your and the baby’s father’s yearly gross income’? Participants will be asked to indicate the income of themselves and their partners in seven categories. The responses will be scored from 1 (lowest income) to 7 (highest income).

*Smoking* by 1 item ‘Do you smoke at present’? with 3 response options: not smoking, sometimes or daily.

*Age of the participant by* 1 item: ‘How many years are you?’. Participants will be asked to fill in number of years.

*Living conditions* by two items. ‘With whom do you live?’ (Spouse/partner, Parents, Parents-in-law, Children, No one, Other) and ‘How many people including you live in your home?’

**Statistical analyses and power analysis**

The estimated sample size is based on effect sizes reported in previous studies that have used BB-glasses as a treatment for sleep-disorders (20, 44). These studies showed strong effects on sleep quality in one group of healthy individuals and one group of persons diagnosed with ADHD. Because sleep problems during late pregnancy may have various hormonal and mechanical influences, we expect a medium effect size of about 0.5 (Cohens d) in the pregnant population. Setting the alpha to .05 (two-tailed), power set to .80, correlation between assessments at .50 reveals that 34 participants are needed minimum to detect statistically significant time (pre vs. post) x group (BB vs. control condition) interaction effects (45).

**Ethical considerations**

All participation will be based on written consent and volunteerism. There are no known side-effects, except of transient headache reported in 5-10 by wearing the blue-blocking glasses. The trial will apply the Regional Ethical Comity for approval to conducting the study. The trial will be registered in the ClinicalTrials.gov database.

**Project team**

The main-supervisor is Professor Ståle Pallesen, Department of Psychosocial Science, Faculty of Psychology, University of Bergen. Pallesen is a Somnologist (European accreditation) and has been PI on several RCTs on CBT-based treatments for sleep disorders, and has long experience within clinical and epidemiological sleep medicine. He has had a leading role in the establishment of a human sleep laboratory at the Faculty of Psychology, UiB.

The co-supervisors are:

Associate professor Janne Grønli, Sleep and Performance Research Center, Washington State University, US and Department of Biological and Medical Psychology, Faculty of Psychology, University of Bergen. She is a Somnologist (European accreditation) and has conducted several studies on sleep and circadian rhythms, in men and rodents. Recently she and professor Pallesen investigated how exposure to 30 minutes of LED light before bedtime affected alertness and sleep quality, subjectively and objectively (16).

Associate professor Roger Ekeberg Henriksen. Department of Health- and Social Sciences, Bergen University College. He is educated nurse, specialized within the field of psychiatry. He has recently defended his PhD-thesis on stress and somatic health in pregnant woman. Henriksen has experience with data collection from a previous study using blueblockers as an intervention and actigraphy as outcome measure. In the present project, he will contribute with the data collection.

The project collaborator is MD/PhD-student Tone Elise Gjøtterud Henriksen. Chief Consultant Psychiatrist at Valen Hospital and a PhD researcher at Section of Psychiatry, Clinical Institute 1, University of Bergen. She has conducted an RCT for measuring the effect of blue-blocking glasses used as ad-on treatment in bipolar mania (26).

**Budget**

The actigraphy equipment is already available as a resource provided by the human sleep lab (lead by the main supervisor) at the Faculty of Psychology and Tone Henriksen at Section of Psychiatry, Clinical Institute, University of Bergen. Henriksen has also BB-glasses available as a resource.

**The following publications will be presented in the thesis:**

Three original papers are planned produced from this project. The papers will all be submitted for publication in high impact, international peer review journals. Results of the present project will also be disseminated on national and international relevant congress/conferences. The proposed titles of the papers are:

1) Light exposure, alertness and sleep pattern in healthy nulliparous women in the third trimester of pregnancy.

2) A randomized trial on the effect of blue-blocking glasses on sleepiness and sleep quality among nulliparous women in third trimester of the pregnancy.

3) A randomized trial on the effect of blue-blocking glasses on melatonin onset, mental health and potential affects among nulliparous women in third trimester of the pregnancy.

Popular scientific paper: “Sleep disturbances in pregnant women – are there any safe treatments?” This paper will be written in Norwegian and will be submitted to the Norwegian journal for midwifes “Tidsskrift for jordmødre”.

**Time plan**

| S2016 | Planning the study, complete the protocol for the trial |
| --- | --- |
| A2016 | Apply to ethical committee (REK), Recruit pregnant women, data collection, analyse the data |
| S2017 | Recruit pregnant women, data collection, analyse the data |
| A2017 | Recruit pregnant women, data collection, analyse the data |
| S2018 | Writing paper 1 |
| A2018 | Writing paper 2 |
| S2019 | Writing paper 3 |
| A2019 | Writing popular scientific paper |
| S2020 | Writing and submitting PhD thesis |

S=spring, A=autumn

**References**

1. Facco FL, Kramer J, Ho KH, et al. Sleep disturbances in pregnancy. Obstetrics and Gynecology. 2010;115(1):77-83.

2. Wilson DL, Barnes M, Ellett L, et al. Decreased sleep efficiency, increased wake after sleep onset and increased cortical arousals in late pregnancy. ANZJOG. 2011;51(1):38-46.

3. Abbott SM, Attarian H, Zee PC. Sleep disorders in perinatal women. Best Pract Res Clin Obstet Gynaecol. 2014;28(1):159-68.

4. Mindell JA, Jacobson BJ. Sleep Disturbances During Pregnancy. JOGNN: Journal of Obstetric, Gynecologic and Neonatal Nursing. 2000;29:590-7.

5. Nodine PM, Matthews EE. Common sleep disorders: management strategies and pregnancy outcomes. J Midwifery Womens Health. 2013;58(4):368-77.

6. Dorheim SK, Bjorvatn B, Eberhard-Gran M. Insomnia and depressive symptoms in late pregnancy: a population-based study. Behav Sleep Med. 2012;10(3):152-66.

7. Sivertsen B, Lallukka T, Salo P, Pallesen S, Hysing M, Krokstad S, et al. Insomnia as a risk factor for ill health: results from the large population-based prospective HUNT Study in Norway. Journal of sleep research. 2014;23(2):124-32.

8. Baglioni C, Battagliese G, Feige B, Spiegelhalder K, Nissen C, Voderholzer U, et al. Insomnia as a predictor of depression: a meta-analytic evaluation of longitudinal epidemiological studies. J Affect Disord. 2011;135(1-3):10-9.

9. Sivertsen B, Salo P, Mykletun A, Hysing M, Pallesen S, Krokstad S, et al. The bidirectional association between depression and insomnia: the HUNT study. Psychosom Med. 2012;74(7):758-65.

10. Morphy H, Dunn KM, Lewis M, Boardman HF, Croft PR. Epidemiology of insomnia: a longitudinal study in a UK population. Sleep. 2007;30(3):274-80.

11. Neckelmann D, Mykletun A, Dahl AA. Chronic insomnia as a risk factor for developing anxiety and depression. Sleep. 2007;30(7):873-80.

12. Ding XX, Wu YL, Xu SJ, Zhang SF, Jia XM, Zhu RP, et al. A systematic review and quantitative assessment of sleep-disordered breathing during pregnancy and perinatal outcomes. Sleep Breath. 2014;18(4):703-13.

13. Palagini L, Gemignani A, Banti S, Manconi M, Mauri M, Riemann D. Chronic sleep loss during pregnancy as a determinant of stress: impact on pregnancy outcome. Sleep medicine. 2014;15(8):853-9.

14. Ram S, Seirawan H, Kumar SK, Clark GT. Prevalence and impact of sleep disorders and sleep habits in the United States. Sleep Breath. 2010;14(1):63-70.

15. Okun ML, Ebert R, Saini B. A review of sleep-promoting medications used in pregnancy. Am J Obstet Gynecol. 2015;212(4):428-41.

16. Gronli J, Byrkjedal Kristiansen I, Bjorvatn B, Nødtvedt Ø, Hamre B, Pallesen S. Reading on an Ipad or in a book in bed– the impact on human sleep. A randomized controlled crossover trial. . Sleep medicine. 2016;In press.

17. Khalsa SB, Jewett ME, Cajochen C, Czeisler CA. A phase response curve to single bright light pulses in human subjects. J Physiol. 2003;549(Pt 3):945-52.

18. Cajochen C. Alerting effects of light. Sleep Med Rev. 2007;11(6):453-64.

19. Bennett S, Alpert M, Kubulins V, Hansler RL. Use of modified spectacles and light bulbs to block blue light at night may prevent postpartum depression. Med Hypotheses. 2009;73(2):251-3.

20. Burkhart K, Phelps JR. Amber lenses to block blue light and improve sleep: a randomized trial. Chronobiol Int. 2009;26(8):1602-12.

21. Berson DM. Phototransduction in ganglion-cell photoreceptors. Pflugers Arch. 2007;454(5):849-55.

22. Brainard GC, Hanifin JP, Greeson JM, Byrne B, Glickman G, Gerner E, et al. Action spectrum for melatonin regulation in humans: evidence for a novel circadian photoreceptor. J Neurosci. 2001;21(16):6405-12.

23. Phelps J. Dark therapy for bipolar disorder using amber lenses for blue light blockade. Med Hypotheses. 2008;70(2):224-9.

24. Kayumov L, Casper RF, Hawa RJ, Perelman B, Chung SA, Sokalsky S, et al. Blocking low-wavelength light prevents nocturnal melatonin suppression with no adverse effect on performance during simulated shift work. J Clin Endocrinol Metab. 2005;90(5):2755-61.

25. Sasseville A, Paquet N, Sevigny J, Hebert M. Blue blocker glasses impede the capacity of bright light to suppress melatonin production.(Author abstract). Journal of Pineal Research. 2006;41:73-8.

26. Henriksen TE, Skrede S, Fasmer OB, Hamre B, Gronli J, Lund A. Blocking blue light during mania - markedly increased regularity of sleep and rapid improvement of symptoms: a case report. Bipolar Disord. 2014;16(8):894-8.

27. World Health Organization. Towards a new Global Strategy for Women's, Children's and Adolescent's Health. BMJ. 2015;351:Suppl.

28. Chen YC, Sheen JM, Tiao MM, Tain YL, Huang LT. Roles of melatonin in fetal programming in compromised pregnancies. Int J Mol Sci. 2013;14(3):5380-401.

29. Hauge ER, Berle JO, Oedegaard KJ, Holsten F, Fasmer OB. Nonlinear analysis of motor activity shows differences between schizophrenia and depression: a study using Fourier analysis and sample entropy. PLoS One. 2011;6(1):e16291.

30. Sivertsen B, Omvik S, Havik OE, Pallesen S, Bjorvatn B, Nielsen GH, et al. A comparison of actigraphy and polysomnography in older adults treated for chronic primary insomnia. Sleep. 2006;29(10):1353-8.

31. Carney CE, Buysse DJ, Ancoli-Israel S, Edinger JD, Krystal AD, Lichstein KL, et al. The consensus sleep diary: standardizing prospective sleep self-monitoring. Sleep. 2012;35:287-302.

32. American Academy of Sleep Medicine. International classification of sleep disorders Darien, IL: American Academy of Sleep Medicine. 2014;3.

33. Milton K, Bull FC, Bauman A. Reliability and validity testing of a single-item physical activity measure. Br J Sports Med. 2011;45: 203-208.

34. Pallesen S, Bjorvatn B, Nordhus IH, Sivertsen B, Hjørnevik M, Morin CM. A new scale for measuring insomnia: the Bergen Insomnia Scale. Percept Mot Skills. 2008;107(3):691-706.

35. Akerstedt T, Gillberg M. Subjective and objective sleepiness in the active individual. Int J Neurosci. 1990;52(1-2):29-37.

36. Watson D, Clark LA, Tellegen A. Development and validation of brief measures of positive and negative affect: the PANAS scales. Journal of personality and social psychology. 1988;54(6):1063.

37. Nicassio PM, Mendlowitz DR, Fussell JJ, Petras L. The phenomenology of the pre-sleep state: The development of the Pre-sleep Arousal Scale. Behav Res Ther. 1985;23(3):263-271.

38. Johns MW. A new method for measuring daytime sleepiness: the Epworth sleepiness scale. Sleep. 1991;14(6):540-5.

39. Beck AT, Epstein N, Brown G, Steer RA. An Inventory for Measuring Clinical Anxiety: Psychometric Properties. Journal of Consulting and Clinical Psychology. 1988;56(6):893-897.

40. Beck AT, Steer RA, Brown GK. Manual for the Beck Depression Inventory-II. San Antonio, TX: Psychological Corporation. 1996

41. Meyer TJ, Miller ML, Metzger RL, Borkovec TD. Development and Validation of the Penn State Worry Questionnaire. Behav Res Ther. 1990;28(6):487-495.

42. Cohen S, Kamarck T, Mermelstein R. A global measure of perceived stress. J Health Soc Behav. 1983;24(4):385-96.

43. Røysamb E, Vittersø J, Tambs K. The Relationship Satisfaction scale-psychometric properties. Norsk Epidemiologi. 2014;24((1/2)):187-94.

44. Fargason R, Preston T, Hammond E, May R, Gamble K. Treatment of attention deficit hyperactivity disorder insomnia with blue wavelength light-blocking glasses. Bipolar disorder. 2013;24(25).

45. Faul F, Erdfelder E, Lang AG, Buchner A. G*Power 3: a flexible statistical power analysis program for the social, behavioral, and biomedical sciences. Behav Res Methods. 2007;39(2):175-91.
